# Gallbladder schistosomiasis

**DOI:** 10.4322/acr.2024.516

**Published:** 2024-10-08

**Authors:** Gabriela Del Angel-Millán, José Jukemura, Júlia Bragion Bicudo, Ricardo Jureidini, André Luís Montagnini, Vanderlei Segatelli, Thiago Costa Ribeiro, Guilherme Naccache Namur, Thiago Nogueira Costa, Lucas Cata Preta Stolzemburg, Emilio Elias Abdo, Ulysses Ribeiro, Paulo Herman, Estela Regina Ramos Figueira

**Affiliations:** 1 Hospital das Clínicas da Faculdade de Medicina da Universidade de São Paulo, Departamento de Gastroenterologia e Nutrologia, Divisão de Cirurgia do Aparelho Digestivo, São Paulo, SP, Brasil; 2 Hospital das Clínicas da Faculdade de Medicina da Universidade de São Paulo, Departamento de Patologia, São Paulo, SP, Brasil

**Keywords:** Schistosomiasis, Gallbladder Diseases, Gallbladder Neoplasms, Parasitic Diseases

## Abstract

Schistosomiasis is an infectious disease caused by parasitic flatworms of the genus Schistosoma. The species *Schistosoma mansoni* is associated with hepatosplenic disease. Schistosomiasis involving the gallbladder alone is highly unusual, with a few cases reported. Herein, we present the case of a woman from a region with endemic schistosomiasis who presented with a painless solid lesion and wall thickening of the gallbladder. She underwent an uneventful laparoscopic cholecystectomy. Microscopic examination of the surgical specimen revealed *Schistosoma mansoni* eggs associated with granulomatous reaction, leading to the diagnosis of schistosomiasis of the gallbladder, prompting subsequent treatment with praziquantel and follow-up. This case illustrates the importance of suspicion for this diagnosis in endemic areas, as it can be misdiagnosed with malignancy if not examined microscopically. Complications and treatment strategies are poorly characterized for the few cases of schistosomiasis; reporting this case can serve as a helpful reminder of a rare presentation of this disease.

## INTRODUCTION

Schistosomiasis is an infectious disease caused by parasitic flatworms of the genus Schistosoma and remains a significant global health concern.^1^ In 2021, 75.3 million people were reported to be treated, while 251.4 million people required preventive treatment; it has been estimated that 230-250 million people are infected annually, with a higher prevalence in tropical, subtropical regions and developing countries with poor access to potable water and adequate sanitization.^2,3^ In endemic areas, schistosomiasis remains a major public health problem affecting approximately 240 million people and resulting in nearly 200,000 deaths each year.^2,4^

People get infected during agricultural, domestic, occupational, and recreational activities involving infested water exposure.^2^ Clinical manifestations can vary from acute to chronic presentations, predominantly affecting the intestinal and urogenital tract. Some species have been related to infection of the liver and spleen.^5^

Infection of the gallbladder is rare. The first description dates from 1949; since then, less than 100 cases have been reported in the English literature.^6^ The clinical implications of this presentation are poorly characterized, and treatment strategies can vary according to the different case reports.^7^ Here, we present a case of a female patient with isolated affection of gallbladder initially suspected to have gallbladder cancer, highlighting the diagnostic and management challenges associated with this uncommon presentation of schistosomiasis.

## CASE REPORT

A 72-year-old woman from the northeast region of Brazil sought medical attention for a two-month history of moderate dyspnea and nonspecific abdominal pain with vague radiation to the upper right quadrant, accompanied by back pain. Her medical history included treated pulmonary tuberculosis and blastomycosis ten years ago. She has been a heavy smoker until 8 years ago.

During the physical examination, faint rales were noted upon auscultation of the left pulmonary base. Abdominal examination revealed no abnormalities, including a negative Murphy’s sign and absence of palpable masses.

**Figure 1:**
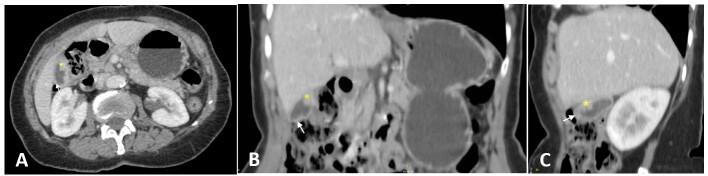
Abdominal Computed Tomography scan shows generalized thickening of the gallbladder wall (white arrows) and the polypoid and solid components (yellow marks) in axial (**A**), coronal (**B**), and sagittal (**C**) planes.

Abdominal ultrasound examination showed diffuse thickening of the gallbladder wall with solid irregular nodules suggesting wall infiltration. The abdominal computerized tomography (CT) revealed normal liver size, portal vein, and bile ducts; the gallbladder showed irregular parietal thickening with a polypoid aspect at the fundus (Figure 1).

The presence of gallbladder wall thickening on imaging, in the absence of symptoms associated with the patient's age and history of smoking, prompted concern for malignancy.

Tumor marker assessment revealed mildly elevated carcinoembryonic antigen of 9.5 ng/mL (reference range <5 ng/mL) but normal alpha-fetoprotein levels, antigen CA 19-9 and antigen CA 125 levels.

**Figure 2:**
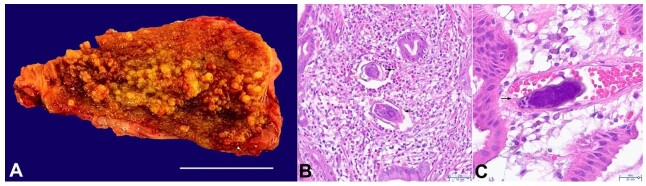
**A –** Macroscopic view of the surgical specimen with the polypoid aspect of the mucosa (scale bar = 4 cm); **B –** Gallbladder mucosa showing *S. mansoni* eggs (black arrows), surrounded by chronic inflammatory infiltrate rich in eosinophils (HE, 20x); **C –** *Schistosoma mansoni* egg with lateral spike is indicated by the black arrow, present in a blood vessel in the gallbladder mucosa (HE, 40x).

Considering the suspicion of a resectable gallbladder cancer, a laparoscopic cholecystectomy (LC) was scheduled with intraoperative frozen section analysis and potential escalation to radical cholecystectomy upon confirmation of malignancy. LC was performed without complications, revealing no intraoperative ascites, hepatic anomalies, or distant lesions. Frozen section examination exhibited no signs of malignancy. The presence of polyps indicated possible hyperplasia or adenoma. The final pathology report was a polypoid lesion measuring 6.8 x 2.5cm, with chronic granulomatous cholecystitis and multiple *Schistosoma mansoni (SM)* eggs distributed throughout the gallbladder's mucosal, submucosal, and serosal layers. Additionally, diffuse hyperplasia of the mucosa with polypoid appearance and chronic granulomatous lymphadenitis with schistosome eggs in the cystic lymph node was observed (Figure 2).

The patient recovered uneventfully and was discharged on the first postoperative day for outpatient follow-up. Given the confirmed diagnosis in the surgical specimen, stool or urine analysis was not pursued. Subsequent treatment with praziquantel was completed. Imaging studies and surgical findings did not reveal liver abnormalities suggestive of fibrosis or portal hypertension. No MRI or liver biopsy was performed thus occult concomitant liver involvement cannot be definitively ruled out.

## DISCUSSION

Schistosomiasis is an ancient disease described in the Egyptian papyrus papers. Theodor Bilharz made the first scientific description in 1851.^8^ Many organs can be affected in schistosomiasis, and the different species are related to the patterns of the disease. The main species that cause hepatobiliary affection are *S. mansoni*, *S. mekongi* and *S. japonicum*.^1,8^

The infection cycle starts when the eggs are excreted from humans in feces. They hatch and mature in the intermediate hosts, freshwater snails. These hosts release motile cercariae into the water, which are the larval stage that penetrate the human skin upon contact with contaminated water. Once in the human body, the cercariae undergo a series of morphological and physiological changes, transforming into the schistosomula. Subsequently, they migrate from the skin in about 5 to 7 weeks, flowing from the blood vessels into the lymphatic circulation, reaching the lung, and reentering the circulation.^9,10^

The schistosomula mature into adult worms, which continue flowing through in the circulation; adult male and female worms mate and produce fertilized eggs; these eggs reach the portal and mesenteric circulation, ending up in organ-specific small blood vessels. The mature eggs then start the immunological reaction in the affected organ.^9,11^

The eggs are highly antigenic and trigger granuloma formation. The antigenic response usually takes about 5-6 weeks. While granulomas in hepatic and intestinal tissues are hallmark features, those in the liver, due to their inability to shed, may progress to fibrosis, contributing to severe disease.^11^

Cases of exclusive involvement of the gallbladder are highly uncommon. In most cases, gallbladder involvement has been associated with advanced hepatic portal fibrosis due to SM. Typical ultrasonographic findings are hyperechogenic wall thickening with external gallbladder wall protuberances. It can potentially be associated with gallbladder fibrosis, which rarely reverses after therapy, and it doesn’t relate to obstruction of the bile duct due to the parasites.^5^

In other case reports, it has been identified as solitary polyps, multiple septations, and calcification in the gallbladder, being identified in CT and magnetic resonance imaging (MRI).^5^ The MRI can provide additional information for diagnosing concomitant liver affection, showing hyperintense signal in T2-weighted sequences, allowing the identification of periportal inflammation.^12^

Due to the few reported cases, there is currently no established diagnostic standard for assessing disease extension. Additionally, there are no reports on the reliability of liver biopsy to confirm involvement in cases with gallbladder schistosomiasis where the liver appears normal in imaging studies. In hepatosplenic schistosomiasis, liver involvement is suspected based on imaging findings and confirmed by Schistosoma ova in feces or urine samples. In cases where these samples do not yield positive results, a liver biopsy may be necessary to establish the diagnosis.^13,14^

In the cases reported in the medical literature, symptoms are vague and mostly absent. Although there have been cases reported as acute cholecystitis, this presentation is unusual; normally, the affection in the gallbladder is chronic, leading to fibrosis, and not be associated with pain or positive Murphy’s sign. Bile duct affection has not been described, and concomitant cholelithiasis is rare.^5^

The diagnosis is challenging since the imaging findings could be present in other scenarios; most cases are misinterpreted as gallbladder malignancy due to the solid components and wall thickening. Similarly, it has also been misdiagnosed as emphysematous cholecystitis and tuberculosis, which are usually found with additional liver fibrosis.^15^

The surgical specimen’s histological examination makes a definitive diagnosis of gallbladder schistosomiasis. Histopathology is characterized by the finding of granulomas containing Schistosoma ova. This highlights the importance of routine analysis of surgical specimens in every case, especially in countries with high rates of schistosomiasis.^16^

In the context of gallbladder schistosomiasis, a small series involving 18 cases of gallbladder disease revealed that Schistosoma ova were detected in stool samples from only 1 patient. Therefore, a negative stool test cannot definitively exclude the diagnosis of schistosomiasis. Confirmation typically relies on identifying Schistosoma ova in surgical specimens from the affected organ. It is also noteworthy that within this series, only 2 patients exhibited involvement of an additional organ concurrent with gallbladder disease at the time of diagnosis.^8^

Treatment typically involves cholecystectomy and antiparasitic therapy with praziquantel. The chronic granulomatous inflammation of the gallbladder may pose technical challenges during surgery; however, the number of cases is low to elucidate if it can bring more complications than other etiologies.^1,17^ Treatment with praziquantel should not be dismissed after the cholecystectomy; this atypical presentation of schistosomiasis highlights different routes of the parasite in the human body and the potential dissemination and affection of other organs, particularly the development of liver fibrosis.^16^

Most common complications are associated with the chronic setting of the infection and the accompanying liver affection, which can lead to periportal fibrosis and secondary portal hypertension. In the described cases of isolated gallbladder involvement, it can lead to the presence of fibrosis and impaired contraction of the gallbladder. In just one case, perforation of the gallbladder was reported.^8,18^

## CONCLUSION

This case emphasizes the importance of considering the diagnosis of gallbladder schistosomiasis in endemic areas and routine histopathological examination of post-cholecystectomy surgical specimens. Systemic treatment is advisable in these cases to mitigate potential complications and disease spread. Reporting such cases aids in understanding clinical presentations and complications and identifying predominant regions of incidence that can guide effective management strategies and public health interventions.
